# An Extended Iranian Family with Autosomal Dominant Non-syndromic Hearing Loss Associated with A Nonsense Mutation in the *DIAPH1* Gene

**DOI:** 10.34172/aim.2023.27

**Published:** 2023-03-01

**Authors:** Marzieh Mohseni, Yusuf Mohammadi, Farzane Zare Ashrafi, Fatemeh Ghodratpour, Khadijeh Jalalvand, Sanaz Arzhangi, Mojgan Babanejad, Mohammad Hossein Azizi, Kimia Kahrizi, Hossein Najmabadi

**Affiliations:** ^1^Genetics Research Center, University of Social Welfare and Rehabilitation Sciences, Tehran, Iran; ^2^Associate Professor of Otolaryngology, Academy of Medical Sciences of IR Iran, Tehran, Iran

**Keywords:** *DIAPH1*, Exome sequencing, Iran, Non syndromic hearing loss

## Abstract

Genetic analysis of non-syndromic hearing loss (NSHL) has been challenged due to marked clinical and genetic heterogeneity. Today, advanced next-generation sequencing (NGS) technologies, such as exome sequencing (ES), have drastically increased the efficacy of gene identification in heterogeneous Mendelian disorders. Here, we present the utility of ES and re-evaluate the phenotypic data for identifying candidate causal variants for previously unexplained progressive moderate to severe NSHL in an extended Iranian family. Using this method, we identified a known heterozygous nonsense variant in exon 26 of the *DIAPH1* gene (MIM: 602121), which led to "Deafness, autosomal dominant 1, with or without thrombocytopenia; DFNA1" (MIM: 124900) in this large family in the absence of *GJB2* disease-causing variants and also OtoSCOPE-negative results. To the best of our knowledge, this nonsense variant (NM_001079812.3):c.3610C>T (p.Arg1204Ter) is the first report of the *DIAPH1* gene variant for autosomal dominant non-syndromic hearing loss (ADNSHL) in Iran.

## Introduction

 Hearing loss (HL) is the most common neurosensory disorder in humans. The incidence rate of congenital HL is estimated to be around 1–2/1000 and 3–4/1000 newborns in developed and developing countries like Iran, respectively.^1^ Non-syndromic hearing loss (NSHL) is highly heterogeneous and accounts for about 70% of hereditary HL.^1,2^ About 20% of NSHL cases have an autosomal dominant mode of inheritance,^1^ and to date, among more than 120 genes identified for NSHL, 51 genes have been assigned to ADNSHL (https://hereditaryhearingloss.org/). From the past to the present, considering the location of Iran in the consanguineous marriage belt and the plentitude of the population with heterogeneous autosomal recessive disorders, many types of research with different techniques have been performed to disclose the genetic etiology of HL in Iran.^1^ In recent years, high throughput sequencing technologies, such as exome sequencing (ES), have drastically increased the efficacy of gene identification in heterogeneous Mendelian disorders such as NSHL, of which the most common genes were *GJB2*, *SLC26A4*, *MYO15A*, *MYO7A*, *CDH23*, and *TMC1 *in Iran.^1-3^

 In this report, we describe the first ADNSHL Iranian family with the known *DIAPH1* (NM_001079812.3):c.3610C > T (p.Arg1204Ter) mutation using the ES technique. The introduction of this family and the process of predicting the disease-causing variant emphasize the importance of genetic counselling for otolaryngologists, careful clinical data collection, and underlying late-onset occurrence in heterogeneous disorders.

## Case Report

**Figure 1:**
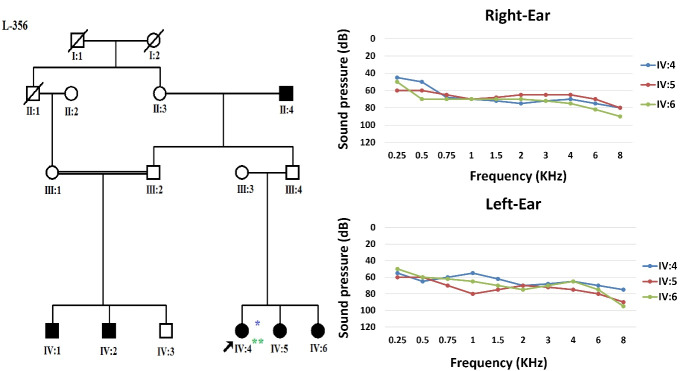
Previous Pedigree of the Family and Pure Tone Audiometry for Left and Right Ears of Patients (*individual run on OtoSCOPE, **individual run on exome sequencing)

 Nearly 20 years ago, an extended consanguineous Iranian family from Semnan, northern Iran, with sensorineural HL and apparently an autosomal recessive pedigree, was referred to the Genetics Research Center (GRC) of the University of Social Welfare and Rehabilitation Sciences (USWR), Tehran, Iran, for unravelling the etiology of HL (Figure 1). After obtaining informed consent, clinical evaluation and family history were completed. The affected members evaluated for physical examination and clinical otologic findings were normal; also, no thrombocytopenia was detected. Three patients were examined for HL using pure-tone audiometry screening and showed progressive moderate to severe NSHL (Supplementary File 1).

 After sampling the peripheral blood of all 13 members of the family, the DNA of the individuals was extracted by standard salting-out protocols.^4^ The DNA quantity and quality were accurately assessed by NanoDrop^TM^ 2000/2000c Spectrophotometers - Thermo Fisher and running on 1.2% agarose gel.

**Figure 2:**
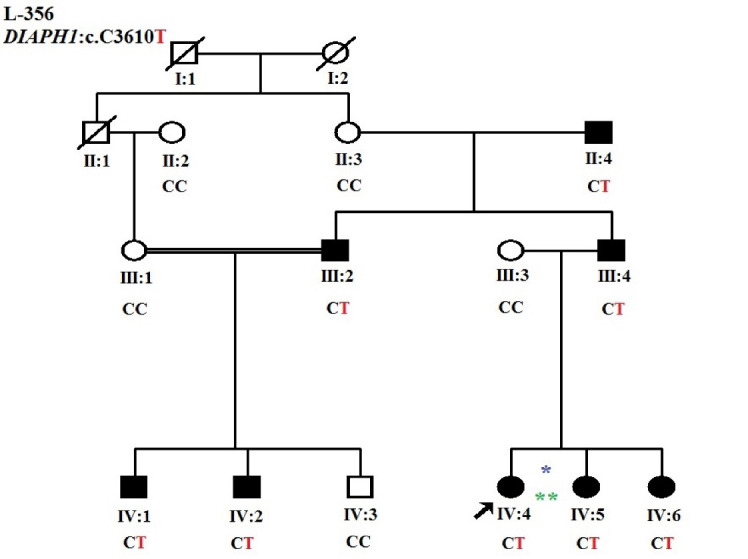
Updated Pedigree with Autosomal Dominant Inheritance (*individual run on OtoSCOPE, **individual run on exome sequencing)

**Figure 3:**
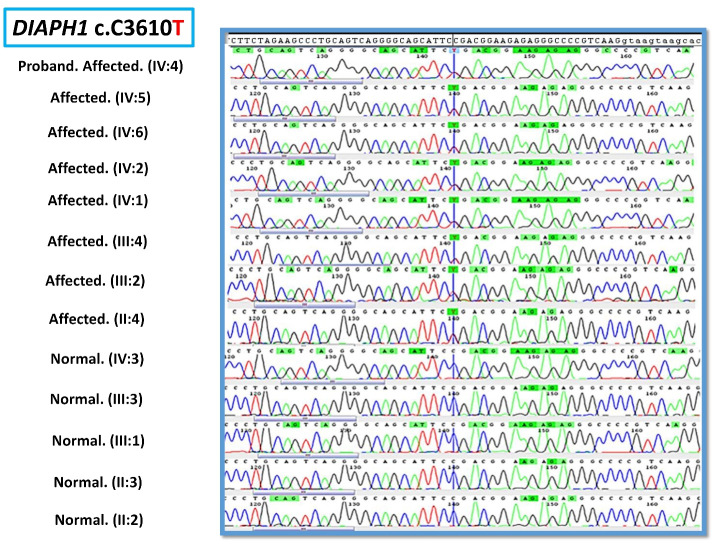
Electropherogram of c.3610C > T in *DIAPH1 *Gene in the Family

 As a first step, *GJB2* screening was performed using Sanger sequencing. Due to the negative results of *GJB2* pathogenic variants, the OtoSCOPE panel (V5&V6),^5^ which covers 89 known deafness-associated genes, including *the DIAPH1 *gene, was used, and the family was OtoSCOPE-negative for ARNSHL. A few years later, the family was re-evaluated by ES (Illumina NextSeq500 using the SureSelectXT Human All Exon V6 kit; Illumina, Inc.). Tertiary analysis was performed with greater focus on homozygous variants and filtering according to the autosomal recessive model. Variant filtering and prioritization were applied using the ACMG guidelines for the interpretation of sequence variants and the following databases and *in-silico* algorithms: gnomAD,^6^ 1000 genomes, Iranome,^7^ OMIM, ClinVar, dbSNP, ExAC Gene Constraints, VS-PolyPhen2, GERP + + , VS-SIFT, and PhyloP. Bioinformatics analysis failed to find a promising homozygous variant in our proband (IV: 4), and just a known heterozygous nonsense variant c.3610C > T in exon 26 of the *DIAPH1* gene was identified that was previously reported as a cause of ADNSHL (Table 1). This prompted us to re-evaluate the family and clinical history. After thorough evaluation, it was revealed that individuals (III: 2) and (III: 4) showed late-onset HL (Figure 2). Confirmation of the detected variant and co-segregation study in the family was performed via Sanger sequencing, resulting in the identification of heterozygous *DIAPH1* (NM_001079812.3):c.3610C > T (p.Arg1204Ter) mutation as the cause of NSHL in the family (Figure 3). The primers used for sequencing of the candidate variant were: Forward primer: 5’-GTGGGAGAGGGGAAATCAAG-3’, Reverse primer: 5’-AACTCAAATCCCTGGGTCCT-3’.

**Table 1:** *In Silico* Prediction of Variant Identified in *DIAPH1* Gene

**Software**	**Mutation taster**	**phyloP20way_mammalian**	**FATHMM**	**LRT**	**CADD-phred**	**phastCons20way_mammalian**	**DANN**	**CADD-raw**	**ACMG Criteria**
Prediction	Disease causing	—	Damaging	Deleterious	—	—	—	—	Pathogenic
Score	0.81	1.048	0.904	0.000012	43	0.995	0.997	13.753	PVS1-PM2

## Discussion

**Figure 4:**
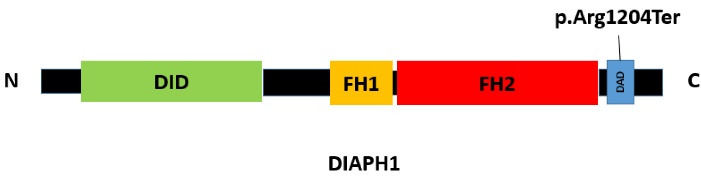
Schematic Illustration of DIAPH1 Protein

 The human diaphanous related formin 1; *DIAPH1* (also known as DFNA1, DIA1), located on 5q31.3, which encodes homodimeric “Protein diaphanous homolog 1” belongs to the human formin family.^8^ This protein includes GBD/FH3 (DID), FH1, FH2 and DAD domains and is involved in actin polymerization and microtubule stability (Figure 4).^8^ Mutations in *DIAPH1* are associated with “Deafness, autosomal dominant 1, with or without thrombocytopenia; DFNA1” (MIM: 124900) and “Seizures, cortical blindness, microcephaly syndrome; SCBMS” (MIM: 616632).^8^

*DIAPH1* plays an essential role in hearing via regulating actin filaments assembly in hair cells in the inner ear of humans. Since identifying *DIAPH1* as the first causative gene of ADNSHL in a large Costa Rican family in 1997, among the reported ones, only a few mutations in this gene have been reported with DFNA1 without thrombocytopenia, and most of them have additional symptoms (DFNA1 + Thrombocytopenia).^8^

 Until now, there has been no report of *DIAPH1* mutations with ADNSHL in any Iranian family. There are only two articles that have been published on the *DIAPH1* gene in Iran; one is the introduction of a rare case with homozygous *DIAPH1* mutation leading to SCBMS,^9^ and the other surveyed the expression patterns of *DIAPH1* and virulence genes of dental pathogenic bacteria in oral squamous cell carcinoma patients.^10^

 Here, we report the first Iranian family with ADNSHL and known nonsense (p.Arg1204Ter) mutation located in the DAD domain near the carboxyl terminus of the DIAPH1 protein. This mutation was first reported by Ueyama et al in two unrelated Japanese families suffering from HL in 2016.^11^ The *DIAPH1* (NM_001079812.3):c.3610C > T (p.Arg1204Ter) mutation leads to early termination preceding the (RRKR^1204-1207^) amino acid motif in the DAD C-terminus and producing constitutively active DIA1 mutant which disrupts the autoinhibitory interaction of DID-DAD domains, and as a result of the continuous activity of the mutant protein, DFNA1 occurs.^11^

 Some reports have suggested that this mutation causes macrothrombocytopenia and autosomal dominant HL through a dominant gain of function mechanism,^8,12-14^ but the affected individuals in our study showed only ADSNHL without any additional symptoms which would be the confirmation of the previous report of ADSNHL in the *DIAPH1*.^11^

 Our procedure of genetic counselling and the puzzling situation in the interpretation of the mode of inheritance of the disease in this family highlights the importance of obtaining accurate information in genetic counselling, which is the first and essential step of diagnosis in each medical procedure. This is the first report of *DIAPH1* (NM_001079812.3):c.3610C > T (p.Arg1204Ter) variant from Iran. This study could be evidence of pathogenicity for the current variant, in addition to the Japanese research.^11^

## Supplementary File

Supplementary file 1. Pure tone audiometry for the left and right ears of three patients in this family.
