# Supplementary material for: An Extended Iranian Family with Autosomal Dominant Non-syndromic Hearing Loss Associated with A Nonsense Mutation in the DIAPH1 Gene
Source: Arch Iran Med. 2023 Mar 1;26(3):176–80. doi: 10.34172/aim.2023.27 (PMC10685723; doi:10.34172/aim.2023.27)
Supplement: Supplementary file 1 — Pure tone audiometry for the left and right ears of three patients in this family. [file aim-26-176-s001.pdf]

Arch Iran Med. March 2023;26(3):176-180

10.34172/aim.2023.27

http://journalaim.com

Supplementary file 1. Pure tone audiometry for the left and right ears of three patients in this family

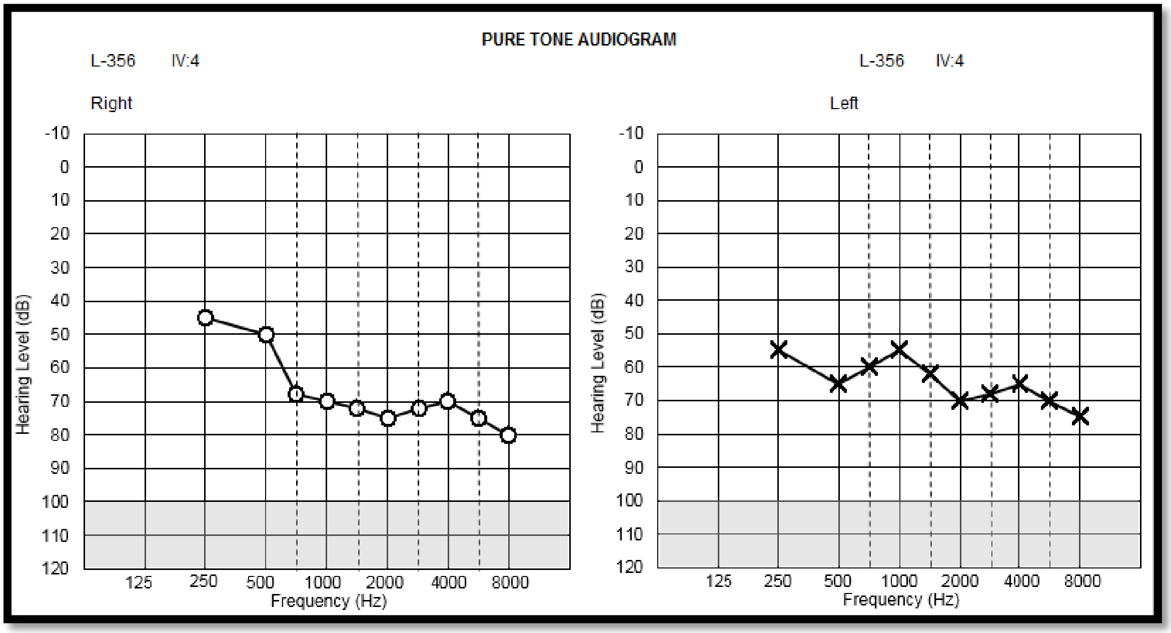

Pure tone audiogram of patient IV: 4

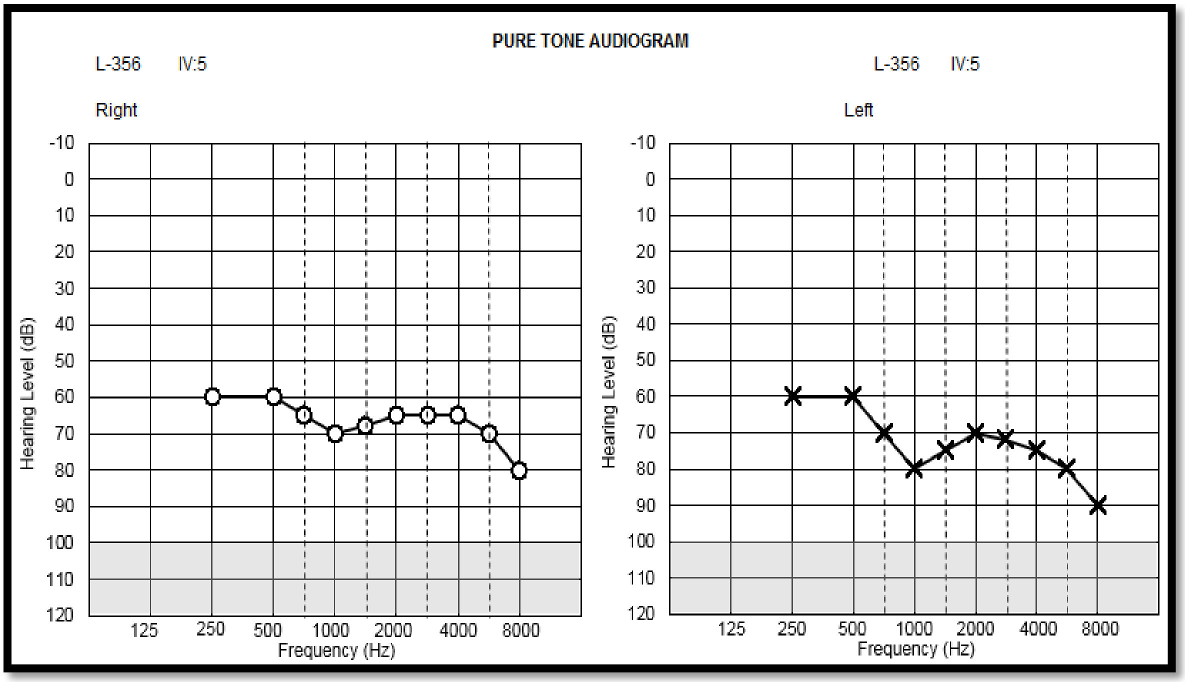

Pure tone audiogram of patient IV: 5

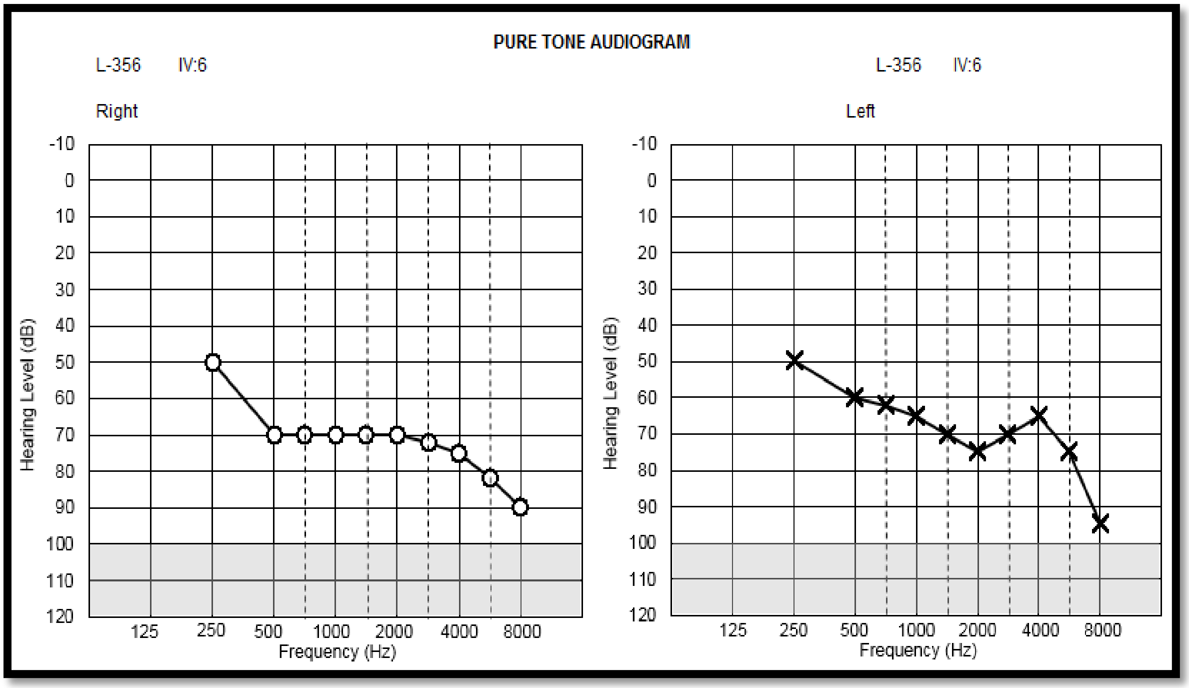

Pure tone audiogram of patient IV: 6
